# Cross over to collective rearrangements near the dry-wet transition in two-dimensional foams

**DOI:** 10.1038/s41598-023-31577-w

**Published:** 2023-03-27

**Authors:** Naoya Yanagisawa, Rei Kurita

**Affiliations:** Department of Physics, Tokyo Metropolitan University, 1-1 Minamioosawa, Hachiouji-shi, Tokyo 192-0397 Japan

**Keywords:** Soft materials, Phase transitions and critical phenomena

## Abstract

Liquid foams respond plastically to external perturbations over some critical magnitude. This rearrangement process is directly related to the mechanical properties of the foams, playing a significant role in determining foam lifetime, deformability, elasticity, and fluidity. In this paper, we experimentally investigate the rearrangement dynamics of foams near a dry-wet transition. When a foam transforms from a dry state to a wet state, it is found that considering collective events, separated T1 events propagate in dry foams, while T1 events occur simultaneously in wet foams. This cross over to collective rearrangements is closely related to the change in local bubble arrangements and mobility. Furthermore, it is also found that a probability of collective rearrangement events occurring follows a Poisson distribution, suggesting that there is little correlation between discrete collective rearrangement events. These results constitute progress in understanding the dynamical properties of soft jammed systems, relevant for biological and material sciences as well as food science.

## Introduction

A liquid foam has a cellular structure composed of a dispersion of air bubbles squeezed against each other. These form a network of liquid thin films which combine at junctions (Plateau borders and nodes)^1–3^. Owing to their complex structure, the typical rheological properties of foams involve elasticity, plasticity, fluidity, and collapse dynamics^4–9^. When an external force is applied to foams, they may respond elastically when the force is small, but they may also flow like liquids when the force is large^10–13^. Using these properties, foams have been applied in a wide range of industrial contexts, such as in cosmetics, pharmaceuticals, biomedical products and daily commodities such as foods, beverages, and detergents. Thus, it is important from a practical perspective to understand the dynamical properties of foams, particularly how they relate to their lifetime, deformability, elasticity, and fluidity.

The physical properties of foams largely depend on their liquid fraction $\phi$^1,2,14,15^. At a jamming transition point $\phi _J$ located at around 0.16 in two-dimensional foams and around 0.36 in three-dimensional foams^16–18^, the bubbles in foams become completely spherical. It is known that static and dynamical critical properties are observed on approaching $\phi _J$^17,19,20^. For example, it has been reported that the mean contact number of bubbles *Z* obeys the relation *Z* $\propto$ $\left( \phi _{J}-{\phi }\right) ^{0.5}$ for $\phi < \phi _J$^21,22^. The dynamics of bubble rearrangements in the coarsening (Ostwald ripening) of foams becomes slower on approaching $\phi _J$^23^. Recently, we experimentally investigated rearrangement dynamics in a two-dimensional wet foam near $\phi _J$ by injecting a constant amount of liquid into the foam. It was found that the rearrangement time and the correlation length associated with the rearrangement of the foam increased as $\phi$ was increased. The nature of the collective bubble rearrangements depends on the size distribution of the bubbles themselves^24^. When the liquid fraction is low, foams are referred to as being in a dry state. In a dry state, the bubble shape is polyhedral and local bubble rearrangement is induced by bubble deformation^25–27^. Meanwhile, bubbles can be rearranged by displacements in a wet foam. The dynamical behavior changes at the dry-wet transition point $\phi _R$^14,15^.

Although the local characteristics of bubbles near the dry-wet transition have been studied, the macroscopic rearrangement dynamics of foams near the dry-wet transition is poorly understood, particularly how the displacements which occur when bubble rearrangements occur can change i.e. it is unclear where and how bubble rearrangements occur during the rearrangement process. The rearrangement process should be directly related to the dynamical properties of soft jammed systems. These include not only foams, but emulsions, concentrated suspensions and colloids as well. Moreover, it is also unclear whether there is a correlation between discrete rearrangement events, that is, whether one rearrangement process induces another rearrangement event nearby, or whether each rearrangement event is independent. The latter point is particularly interesting in its connection with avalanches of rearrangement events in foams and amorphous systems^28–32^. Thus, in this work, we experimentally investigate the dynamics of the structural rearrangement of foams near the dry-wet transition.

## Results

**Figure 1:**
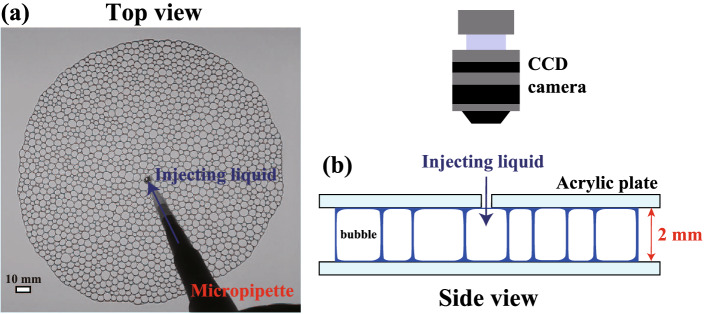
(**a**) and (**b**) Schematic of the experimental setup from the top and side, respectively. We slowly inject a solution (700 μl) into a foam from the small hole pierced in the center of the acrylic plate using a micropipette. $\phi _{2D}$ increases by about 1% as a result. We then observe the rearrangement from the top of the foam using a CCD camera. After the rearrangement completes, we add solution again and investigated the $\phi _{2D}$ dependence of the rearrangement dynamics.

Firstly, we briefly describe the experimental setup in Fig. 1. We place a foam on an acrylic plate and cover it with another acrylic plate. The foam consists of a monolayer of bubbles and is considered quasi two-dimensional. In our experiment, we prepared a nominally polydisperse foam with a mean bubble diameter of 3.6 ± 0.9 mm and a size distribution of 0.25. We estimated a two-dimensional liquid fraction $\phi _{2D}$, which corresponds to the two-dimensional liquid fraction in a cross section through the center of the sample. Under these conditions, the transition between the dry foam and the wet foam is $\phi _{2D} \sim 0.045$, judging from the bubble mobility^15^. More details are given in the “Materials and methods” section.

In order to observe the rearrangement of the foam, we injected a constant amount of additional solution (700 μl) as a perturbation from a small hole pierced in the center of the sample cell, as shown in Fig. 1a,b. By injecting the solution into the foam, the liquid fraction $\phi _{2D}$ increases by about 1%. As depicted in our previous study^24^, the state of the foam before injection is in a basin of the energy landscape. This is a steady state in the absence of any perturbation since thermal energy can be neglected. The energy landscape is changed by injecting the solution, inducing the foam to relax into a new basin close to the initial state, a process which we can observe^24^. After the rearrangement is completed, we added solution again, observing the rearrangement process for different $\phi _{2D}$. In this way, we investigated the liquid fraction dependence of the rearrangement dynamics sequentially. This sequence was performed 10 times. We note that the bubble rearrangement occurs over several minutes, while the coarsening of the bubbles via T2 event occurs over several hours^2^. The timescale for the drainage of the wet foam (the time for the liquid in the foam to flow out) is about an hour, which is larger than the timescale of our observations (about a few minutes). Thus, we consider that the influence of the drainage is small in our experiment. Thus, the coarsening dynamics does not affect the rearrangement dynamics. We note here that applying a mechanical perturbation such as compression of shear is general in the context of rheology^33,34^. However, in experimental studies on jamming systems such as granular materials, it is difficult to combine shear and actual observations. Instead of shear, local perturbations such as inflating the particles are sometimes applied^35^.

Firstly, we show the rearrangement process for $\phi _{2D} = 0.034$, as shown in Supplementary movie 1. The bubbles near the center of the foam flow and subsequently return to their original positions immediately after the injection^24^. This is followed by several large collective rearrangements. Here, we define a time $t_s$ after the injection when the maximum displacement $u_{max}$ of the bubbles at the center of the foam within a time interval of 1 s becomes smaller than the spatial resolution (0.21 mm)^24^. The effect of the injection is considered to be negligible after $t_s$. We find that $t_s$ for $\phi _{2D}$ = 0.019, 0.042, and 0.066 are 6 s, 14 s, and 20 s respectively. Although the injected volume of solution and injection speed both affect $t_s$, the dynamics of the foam after $t_s$ is insensitive to $t_s$, that is, the dynamics after $t_s$ only reflects the rearrangement dynamics induced by the change in the energy landscape. The focus of this work is on the rearrangement dynamics after $t_{s}$.

**Figure 2:**
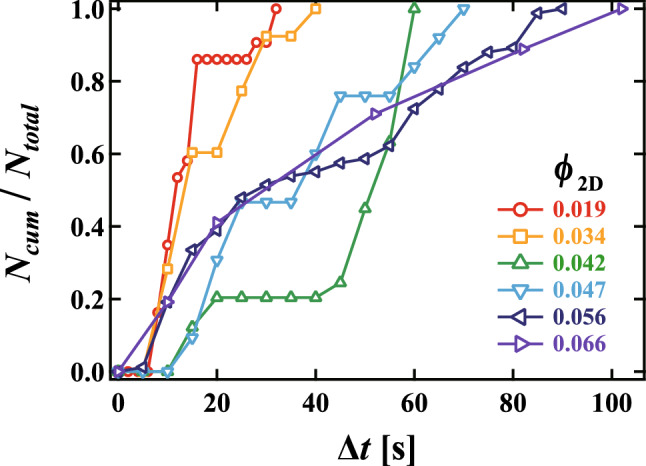
The cumulative number of rearranged bubbles $N_{cum}/N_{total}$ during the rearrangement process as a function of time $\Delta t = t - t_{s}$, where $N_{total}$ is the total number of rearranged bubbles during the rearrangement process and $t_{s}$ is the time when the effect of the solution injection becomes negligible. Red, orange, green, light blue, navy blue and purple lines correspond to $\phi _{2D}$ = 0.019, 0.034, 0.042, 0.047, 0.056 and 0.066, respectively. It is found that $N_{cum}$ increases discontinuously for $\phi _{2D}$ = 0.019, 0.034, 0.042 and 0.047, whereas $N_{cum}$ increases continuously for $\phi _{2D}$ = 0.056 and 0.066.

Here, to quantify the rearrangement of bubbles, we define a bubble as “rearranged”when there is a change in the bubbles it is in contact with. Firstly, we count the number of rearranged bubbles during a rearrangement process. Figure 2 shows the cumulative number of rearranged bubbles $N_{cum}$ as a function of time $\Delta t = t - t_s$ normalized by the total number of rearranged bubbles $N_{total}$. Red, orange, green, light blue, navy blue and purple lines correspond to $\phi _{2D}$ = 0.019, 0.034, 0.042, 0.047, 0.056 and 0.066, respectively. For lower $\phi _{2D}$ or the dry foam, it was found that $N_{cum}$ increases discontinuously, that is, $N_{cum}$ stays in plateaux before sharply increasing and plateauing again. This suggests that rearrangements occur intermittently (see Supplementary movie 1). Meanwhile, in the wet foam at $\phi _{2D}$ = 0.066, $N_{cum}$ increases gradually and continuously. As shown in Supplementary movie 2, the bubbles move continuously.

**Figure 3:**
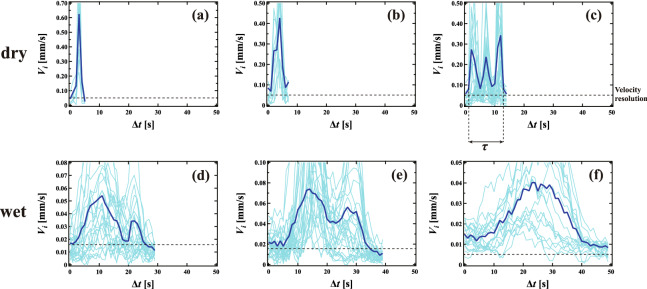
The time evolution of the velocity of the bubbles $V_i$ involved in a single collective rearrangement event for $\phi _{2D}$ = (**a**) 0.019, (**b**) 0.034, (**c**) 0.042, (**d**) 0.047, (**e**) 0.056, and (**f**) 0.066, respectively. The dark blue line shows the mean velocity. We define $\tau$, the characteristic time of a single collective rearrangement event, as the time from the start to the end of the sequence of peaks as shown in (**c**).

To investigate the characteristic collective rearrangement behavior observed during the rearrangement process, we observed the rearrangement in situ. When $\phi _{2D} = 0.042$, it was observed that separated T1 events propagate, as shown in Supplementary movie 3. Here, we compute the velocity of bubble *i* $V_i = \left| \overrightarrow{r_{i}}\left( t+{\Delta }t\right) -\overrightarrow{r_{i}}\left( t\right) \right| /{\Delta }t$, where $\overrightarrow{r_{i}}\left( t\right)$ is the displacement vector of bubble *i* at time *t* and ${\Delta } t$ is a time interval. Figure 3a–f show the time evolution of $V_i$ for $\phi _{2D}$ = 0.019, 0.034, 0.042, 0.047, 0.056 and 0.066, respectively. In order to observe displacements which were large enough to resolve, we chose the time interval $\Delta t$ = 1 s for $\phi _{2D}$ < 0.045, $\Delta t$ = 3 s for 0.045 < $\phi _{2D}$ < 0.065 and $\Delta t$ = 10 s for $\phi _{2D}$ > 0.065, where ${\Delta } t$ is approximately 1/10 of a single collective rearrangement event. We note that the results are insensitive to the choice of $\Delta t$. The resolution of our measurements of $V_i$ for each system is indicated by the black horizontal dashed line. Note that we only show $V_i$ for bubbles involved in the rearrangement events (light blue lines). Dark blue lines correspond to the mean velocity of the rearranged bubbles. For the dry foam, when $\phi _{2D} < 0.045$, it was found that sharp peaks over short time intervals can be observed. Each peak corresponds to a rearrangement via T1 events; for the event at $\phi _{2D} = 0.042$ in the figure, we can see three separated T1 events occurring. The prevalence of this kind of rearrangement, where separated T1 events occur, allows us to classify this as a separated collective rearrangement mode. Meanwhile, for the wet foam, when $\phi _{2D} > 0.045$, the shape of the peaks becomes broader as shown in Fig. 3e,f. This is consistent with the increase in $N_{cum}$ shown in Fig. 2. We do the same for this qualitatively different behavior and classify this as a simultaneous collective rearrangement mode. Our observations suggest that the collective rearrangement mode changes as one goes through the dry-wet transition point, from separated T1 events to simultaneous T1 events.

**Figure 4:**
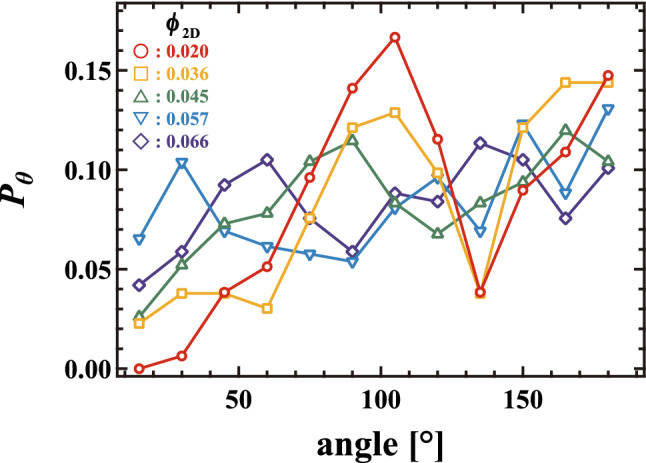
Probability distribution $P_{\theta }$ of the angle between displacement vectors of bubbles in contact in the collective rearrangement events. Red circle, orange square, green triangle, light blue reversed triangle and navy blue diamond symbols correspond to average $\phi _{2D}$ = 0.020, 0.036, 0.045, 0.057, and 0.066, respectively. For dry foams, $\phi _{2D} \le 0.045$, $P_{\theta }$ has peaks at $\theta$ = $90^\circ$ and $180^\circ$. Meanwhile, for wet foams, the shape of the peak becomes broad.

To confirm the existence of a cross over, we calculate the angle $\theta$ between displacement vectors for bubbles in contact. Figure 4 shows the probability distribution $P_{\theta }$ observed during a single collective rearrangement event. Red circles, orange squares, green triangles, light blue reversed triangles, and navy blue diamonds in Fig. 4 correspond to $P_{\theta }$ at $\phi _{2D}$ = 0.020, 0.036, 0.045, 0.057, and 0.066, respectively. For dry foams, $P_{\theta }$ has peaks near $\theta$ = 90$^{\circ }$ and 180$^{\circ }$, corresponding to bubble motion in a typical T1 event. Meanwhile, for wet foams, the peak becomes broader, meaning that the bubbles move randomly. This random motion is consistent with the collective rearrangement dynamics in two-dimensional wet foams reported in Ref.^24^. Thus, we are able to confirm that the motion of bubbles is qualitatively different depending on the liquid fraction.

**Figure 5:**
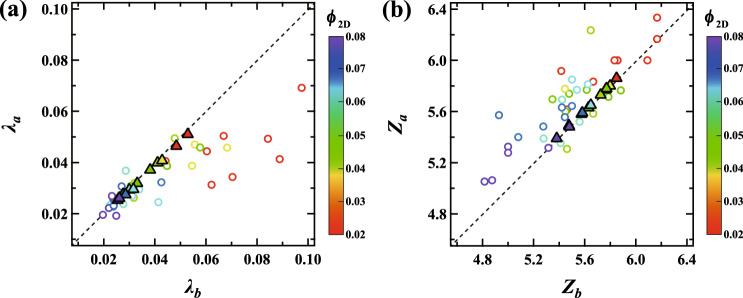
(**a**) Scattering plot of $\lambda _b$ and $\lambda _a$, the mean of $\lambda _i$ averaged over the bubbles involved in a single collective rearrangement event before and after a rearrangement event, respectively. (**b**) Scattering plot of $Z_{b}$ and $Z_{a}$, the mean contact number $z_{i}$ of bubbles involved in a collective rearrangement event before and after a rearrangement, respectively. Filled triangles in (**a**) and (**b**) show the mean $\lambda _{i}$ and $z_{i}$ over all bubbles in the foam, respectively. The black dashed lines represent the lines where $\lambda _b$ and $\lambda _a$ or $Z_{b}$ and $Z_{a}$ are equal. The color of each symbol represents the liquid fraction $\phi _{2D}$.

Next, to clarify the relationship between the rearrangement dynamics and the local internal structure of the foam, we computed the anisotropy of the bubbles. Here, we use a parameter $\lambda _i$ to characterize the anisotropy of bubble *i*^14^; $\lambda _i = \sqrt{\frac{1}{n}\sum \nolimits _{j=1}^{n}\left( r_{j}-\bar{r}\right) ^2}$, where *j* is a pixel at the edge of the bubble, *n* is a total number of pixels *j*, $r_j$ is the distance between the center of bubble *i* and pixel *j* and $\bar{r}$ is the mean of $r_j$. $\lambda _i$ = 0 when the bubble *i* is circular, whereas $\lambda _i>$ 0 when the bubble is anisotropic. We compute the mean of $\lambda _i$ averaged over the bubbles involved in a single collective rearrangement event. $\lambda _b$ and $\lambda _a$ are the mean $\lambda _i$ before and after the rearrangement event, respectively. Figure 5a shows the relationship between $\lambda _b$ and $\lambda _a$; the color of the symbols reflect the liquid fraction as above. The black dashed line represents the line where $\lambda _b$ and $\lambda _a$ are equal. If $\lambda _b$ is larger than $\lambda _a$ (lower than the black line), $\lambda$ becomes smaller during a single collective rearrangement event. We find that $\lambda _b > \lambda _a$ for the dry foam ($\phi _{2D}$ < 0.045) while $\lambda _b \sim \lambda _a$ for the wet foam. This means that for dry foams, the bubbles tend to have a larger anisotropy and the separated T1 events act to reduce this, while for wet foams, the anisotropy starts small and remains almost unchanged during simultaneous rearrangement. We also measured the mean $\lambda _i$ for all bubbles in the foam shown as filled triangle symbols. It was found that the mean of $\lambda _i$ for all bubbles remains almost unchanged for any $\phi _{2D}$. This suggests that the rearrangement occurs where the structure is locally distorted.

We also compute the mean contact number averaged over the bubbles involved in a single rearrangement event. Figure 5b shows the relationship between $Z_b$ and $Z_a$, the mean contact number before and after the rearrangement, respectively. The black dashed line represents the line where $Z_b$ and $Z_a$ are equal. If $Z_b$ is smaller than $Z_a$ (above the black line), *Z* becomes larger during the single collective rearrangement event. We find that $Z_b < Z_a$ for all $\phi _{2D}$; especially for wet foams ($\phi _{2D}>$ 0.045), it seems that $Z_b$ is smaller than $Z_a$ as well as the mean $z_i$ over all bubbles (filled symbols). This means that simultaneous rearrangements occur in regions where the bubbles have smaller contact number. We also note that for the dry foam, the bubble distortion is large when the contact number is small; it seems that separated T1 events do not simply lead to a relaxation of the distortion of bubbles, but simultaneously to growth in *Z*.

**Figure 6:**
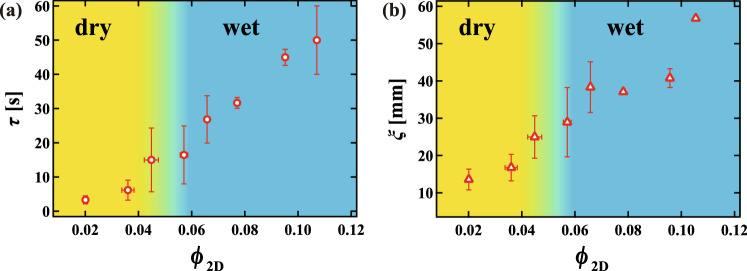
(**a**) The rearrangement time $\tau$ and (**b**) the correlation length $\xi$ of macroscopic rearrangement events averaged over several collective rearrangements as a function of $\phi _{2D}$. It is found that both $\tau$ and $\xi$ increase continuously as $\phi _{2D}$ increases, that is, there is no sign of the dry-wet transition.

We may also quantitatively characterize structural relaxation in the foam by considering characteristic times and length scales. As shown in Fig. 3c, we define the characteristic time for a single collective rearrangement event $\tau$ as the time from the start to the end of the series of sequential peaks in $V_i$. We also define the characteristic length $\xi$ of a single collective rearrangement event as the distance between the two rearranged bubbles which are furthest apart in a series of collective events, as indicated by the double-headed arrow in Fig. S1. Figure 6a,b show $\tau$ and $\xi$ averaged over several (> 5) collective events as a function of $\phi _{2D}$, respectively. We find that both $\tau$ and $\xi$ increase continuously as $\phi _{2D}$ increases. There is no sign of the dry-wet transition, unlike that seen in the mode of rearrangement. In previous work, it was reported that $\tau$ and $\xi$ show critical behavior near the jamming transition point^24^. It was also reported that the number of bubbles involved in a rearrangement event increases with increasing liquid fraction in simulations of 2D foams using the PLAT software^36^, and it continuously increases near the dry-wet transition. Thus, $\tau$ and $\xi$ are determined by the nature of the jamming transition itself, that is, by the change of the energy landscape, not by dynamical factors. This is consistent with the fact that the rearrangement occurs where the distortion of the bubbles is locally significant, as shown in Fig. 5.

**Figure 7:**
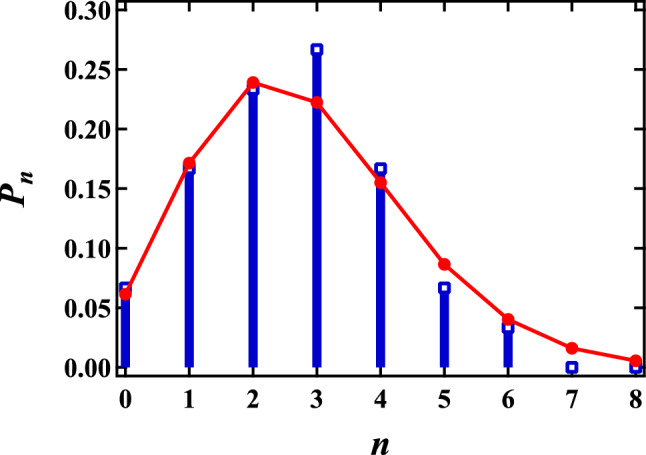
Probability distribution $P_n$ of *n* collective rearrangement events occurring over 5 $\tau$ for all foam samples (blue symbol). The red circles correspond to a fit with a Poisson distribution $P\left( n\right) = {\mu ^{n}e^{-\mu }}/{n!}$, where $\mu$ is a fitting parameter. We obtain $\mu$ = 2.79.

Furthermore, avalanche events in foams and amorphous systems have interested recently^28–32^. Then we computed the correlation between discrete collective rearrangement events. During the rearrangement process, discrete collective rearrangement events occur several times. If there is little correlation between the discrete collective rearrangement events, their frequency over a given time is expected to follow a Poisson distribution. Here, we counted the number of discrete collective rearrangement events *n* within a time interval 5 $\tau$ from $t_s$. Figure. 7 shows the probability $P_n$ of *n*, shown as blue squares. The data contains rearrangement events for all liquid fractions, sampled over 50 discrete collective rearrangement events. We fit $P_n$ with a Poisson distribution $P\left( n\right) = {\mu ^{n}e^{-\mu }}/{n!}$; the red circles correspond to the fit, where $\mu$ = 2.79. As shown in Fig. 7, $P_n$ is well described by a Poisson distribution. Given the properties of a Poisson distribution, it seems that discrete collective rearrangement events are independent of each other.

Finally, we investigate the rearrangement in different foams to confirm how robust the cross over is. Firstly, we observed the rearrangement when the shape of the entire foam is elliptical. The surface tension on the outside of the foam is anisotropic with the entire foam stretched in one direction. It is found that the cross over to collective rearrangements (separated T1 events to simultaneous rearrangement events) is again observed at the dry-wet transition. We also find that $\tau$ and $\xi$ in the elliptical systems are almost the same as those in the circular system (see Supplementary Information). According to Ref.^14^, the distortion of the bubbles is localized in dry foams, meaning that the situation inside the foam is independent of the shape of the foam as a whole. This is consistent with the fact that rearrangement occurs where the internal structure is locally distorted, as shown in Fig. 5.

Here, CHARMY is not typical surfactant, thus we also investigate the different surfactant case by using a 3 wt% solution of ionic surfactant TTAB in glycerol and deionized water. We observed collective rearrangements; separated T1 events propagate in dry foams while T1 events occur simultaneously in wet foams. It is similar to the CHARMY solution case (see Supplemental movies S5 and S6). It was also found that for $\phi _{2D}=0.042$, $\tau$ and $\xi$ are 11 s and 21 mm, respectively, which are little different from the case of the CHARMY solution as shown in Fig. 6.

Here the wall friction could be affected in a quasi-two dimensional experiment^37^. Then we also observed the rearrangement in foams containing several bubbles in the thickness direction of the sample cell. It is found that considering collective events, separated T1 events occur at $\phi$ = 0.13 (see Supplemental movie S7), while simultaneous T1 events occur at $\phi$ = 0.28 (see Supplemental movie S8). Note that the dry-wet transition in 3D should be different from that in 2D. It was reported that the three-dimensional liquid fraction $\phi$ in the sandwiched foam can be estimated from $\phi _{2D}$; this results in a dry-wet transition located around $\phi \sim 0.15$^15^. This is again consistent with the picture of a cross over to collective rearrangements occurring at the dry-wet transition point. We note that the drainage can not neglect in the case of a truly 3D foam with large bubble sizes and the drainage may affect collective rearrangements.

Referring to the previous study, the scaling law between the rearrangement time and the osmotic pressure was proposed^23^. Here we also compared our results with the scaling law. The osmotic pressure $\Pi$ is induced by the contact between the negative pressure at the Plateau boarder and zero pressure at the bubble contact surface. Thus, we roughly estimated $\Pi$ as $\Pi$ = 2${\alpha }{\gamma }/r$, where $\alpha$ is the ratio of the bubble contact surface area to the bubble total surface area, $\gamma$ is the surface tension of the solution and *r* is the radius of the curvature of the Plateau border. For $\phi$ = 0.13 (dry), we obtained $\alpha$ = 0.64, *r* = 0.3 mm, $\Pi$ = 107 Pa and $\tau$ = 3 s. Normalized $\Pi$′ = $\Pi /\left( \gamma /d\right)$ and *T*′ = ${\gamma }\tau /\left( {\eta }d\right)$ ($\gamma$ = 25 mN/m, the averaged diameter of the bubbles *d* = 1 mm, the viscosity of the solution $\eta$ = 0.034 Pa s) are $\Pi$′ = 4.3 and *T*′ = 2200, respectively. As similarly, for $\phi$ = 0.28 (wet), we obtained $\Pi$′ = 2.7 and *T*′ = 3600. It was found that these values are slightly larger, but still consistent with the values reported in the Reference^23^. In addition, the rearrangement reported in Ref.^23^ was induced by the coarsening of the bubbles. Since the time scale was approximately consistent with our results, the cross over to the collective rearrangements can be expected when the rearrangements occur not only by the injection, but also by the coarsening.

## Discussion

Here we discuss the effects of the surface rigidity (viscosity). A previous study reported that the liquid of Plateau borders moves inertially when the bubbles burst in the case of CHARMY and TTAB^9^. In addition, as mentioned above, the rearrangement dynamics in our present study is approximately consistent with the scaling law reported in Ref.^23^, where surfactants with lower surface rigidity was used. Thus, we consider that the surface rigidities of CHARMY and TTAB are small although direct measurement of the surface viscosity is currently difficult.

Next, we discuss the rheology by shear. Some previous studies reported that many T1 events occur in the localized stress regions induced by shear^33,34^. Considering that $\lambda _i$ corresponds to the stress of the bubble, our results that the rearrangements occur in bubbles with larger $\lambda _i$ in dry foams are consistent with these previous studies. Meanwhile, no special feature in shear modulus is observed at $\phi$ = 0.15 reported in Ref.^38^. The shear modulus was averaged over a measurement time by ordinary rheological measurements. Since the collective rearrangements observed in our study are rare events, the effect of the collective rearrangements was not detected. However, it is not obvious whether the rearrangement induced by injecting liquid corresponds to the rearrangement induced by shear. Thus, we need to clarify them in the future.

Finally, we outline the nature of the dry-wet transition from both the view of static and dynamic properties. It was found that the correlation length of the rearrangement $\xi$ is continuous and that the $\phi _{2D}$ dependence of $\xi$ follows critical behavior near the jamming transition. Furthermore, the rearrangement occurs where the distortion of bubbles is large. From this, we conclude that the origin of the rearrangement comes from the nature of the jamming transition. Meanwhile, the dynamical properties change sharply at the dry-wet transition. In a dry foam, when a bubble in a foam is collapsed, other bubbles close to the collapsed bubble are found to be stretched; on the other hand, in a wet foam even just above the dry-wet transition, it can be seen that positional rearrangements occur instead^15^. This local mobility difference induces the difference in macroscopic collective rearrangement events, either separated T1 events or simultaneous T1 events. Thus, it is confirmed that the dry-wet transition is not a static structural transition, but a dynamical transition. Finally, we note that bubbles in the foam are too large to be sensitive to thermal fluctuations. Thus, collective rearrangement events are rare, and the probability of events occurring follows a Poisson distribution. It may also be interesting to investigate the probability of rearrangement events in thermal jamming system, for a more general understanding of the dynamics of soft jammed systems and its relevance for the biosciences, food sciences, and material sciences.

## Conclusions

To summarize, we experimentally investigated the rearrangement dynamics of foams near a dry-wet transition. We observed a cross over in collective rearrangement dynamics, from separated T1 events to simultaneous T1 events going from the dry state to the wet state. In the dry state, separated T1 events occur where bubbles have the large anisotropies of a typical T1 event; in contrast, in the wet state, simultaneous T1 events occur in regions where bubbles have a small contact number. In addition, it is found that the rearrangement time and the correlation lengths associated with discrete collective rearrangement events both increase continuously with liquid fraction, with no sign of a discontinuous transition. This suggests that the origin of the rearrangement events comes from the static structure; however, the dynamics of the rearrangement strongly depends on the mobility of the bubbles in the foam. Furthermore, it is revealed that the probability of collective rearrangement events occurring follows a Poisson distribution, suggesting that discrete collective rearrangement events are independent of each other. These results promise to progress our understanding of soft jammed systems, as seen in the biosciences, food sciences, and material sciences.

## Materials and methods

We used an aqueous solution of household detergent (CHARMY, Lion Co., Japan), diluted to 10% with deionized water. The density of the solution is 1.016 g/cm${^3}$. The surface tension of the solution is 25 mN/m. The viscosity of the solution is 0.034 Pa s. The CHARMY household detergent contains several surfactants (e.g. sodium tetradecenesulfonate, polyoxyethylene fatty acid alkanolamide, alkylamine oxide, sodium alkyl ether sulfate and polyoxyethylene alkyl ether.) In order to investigate the difference in behavior due to the difference in the surfactant type, we used a 3 wt% solution of ionic surfactant TTAB (tetradecyl trimethyl ammonium bromide) in glycerol and deionized water. The density, surface tension and viscosity of the solution are 1.041 g/cm${^3}$, 37 mN/m, 0.020 Pa s, respectively. All experiments use the CHARMY solution unless otherwise stated. We create foams using a capillary glass tube connected to a syringe pump. In this method, it is known that nozzle size and gas flow rates determine the bubble size^24,39^. We fix the inner diameter of the nozzle to 0.4 mm and create the foams by varying the pump speed from approximately 10 ml/min to 70 ml/min to adjust the size distribution of the bubbles. For this study, the mean diameter of the bubbles is 3.6 mm and its standard deviation is 0.9 mm. The total number of the bubbles in each foam is about 1500. The sample thickness is set to 2 mm using a spacer. We take videos of the rearrangement process after liquid injection using a CCD camera (Panasonic, HC-VZX2M) at 1 frame per second using a birds-eye view. We examine static and dynamical properties using an image analysis technique developed in-house. The spatial resolution of the image is 0.21 mm, but the resolution of the center of the bubbles can be estimated to an accuracy of $0.21/\sqrt{N_{diameter}} \approx 0.05$ mm, where $N_{diameter}$ is the number of pixels corresponding to the diameter of the bubbles ($\sim$ 18). We compute a two-dimensional liquid fraction $\phi _{2D}$ using image analysis and $\phi _{2D} = 1 - S_{bubble}/S_{foam}$, where $S_{bubble}$ is the area of bubbles including the interface area, and $S_{foam}$ is the whole area of the foam^15^. $\phi _{2D}$ corresponds to the two-dimensional liquid fraction in a cross section through the center of the sample. We also compute a three-dimensional liquid fraction $\phi$. We directly measure the mass of liquid *m* and the area of the cross section of the foam $S_{foam}$. Then, the liquid fraction $\phi$ is obtained using $\phi$ = $V_{liquid}/V_{foam}$ = $m/{\rho }hS_{foam}$, where $V_{liquid}$, $V_{foam}$, $\rho$, and *h* are the volume of liquid, the volume of the foam, the density of the liquid, the sample cell thickness, respectively. In order to investigate the influence of the friction with the sample boundaries, we use the foam containing several bubbles in the thickness direction of the sample cell. The mean diameter of bubbles in the foam is about 1 mm.

## Supplementary Information

Supplementary Information.

Supplementary Video 1.

Supplementary Video 2.

Supplementary Video 3.

Supplementary Video 4.

Supplementary Video 5.

Supplementary Video 6.

Supplementary Video 7.

Supplementary Video 8.

## Data Availability

All data generated or analyzed during this study are included in this published article and its supplementary information files.
