# Supplementary material for: Cross over to collective rearrangements near the dry-wet transition in two-dimensional foams
Source: Sci Rep. 2023 Mar 27;13:4939. doi: 10.1038/s41598-023-31577-w (PMC10042865; doi:10.1038/s41598-023-31577-w)
Supplement: Supplementary file 1 — Supplementary Information. [file 41598_2023_31577_MOESM1_ESM.pdf]

Supplementary Information for

"Cross over to collective rearrangements near the dry-wet transition in two-dimensional foams"

Naoya Yanagisawa and Rei Kurita
Department of Physics, Tokyo Metropolitan University, Tokyo 192-0397, Japan

(Supplementary movie 1) Movie of the relaxation process for ϕ_{2D} = 0.034.

Playback is at 10x speed.

(Supplementary movie 2) Movie of the relaxation process for ϕ_{2D} = 0.066.

Playback is at 25x speed.

(Supplementary movie 3) Movie of a single collective rearrangement event seen during the relaxation process for ϕ_{2D} = 0.042.

It is found that separated T1 events propagate. Playback is at 5x speed.

(Supplementary movie 4) Movie of a single collective rearrangement event seen during the relaxation process for ϕ_{2D} = 0.066.

It is found that T1 events occur simultaneously. Playback is at 10x speed.

(Supplementary movie 5) Movie of a single collective rearrangement event seen during the relaxation process for ϕ_{2D} = 0.037 in the case of the TTAB solution.

It is found that separated T1 events propagate. Playback is at 2x speed.

(Supplementary movie 6) Movie of a single collective rearrangement event seen during the relaxation process for ϕ_{2D} = 0.057 in the case of the TTAB solution.

It is found that T1 events occur simultaneously. Playback is at 5x speed.

(Supplementary movie 7) Movie of a single collective rearrangement event seen during the relaxation process in a three-dimensional foam for ϕ = 0.13.

It is found that separated T1 events propagate. Playback is at 2x speed. The foam contains several bubbles in the sample cell thickness direction. The liquid fraction ϕ is obtained using $\phi = V_{liquid} / V_{foam} = m / \rho h S_{foam}$, where V_{liquid}, V_{foam}, m, ρ, h, and S_{foam} are the volume of liquid, the volume of the foam, the mass of liquid, the density of the liquid, the sample cell thickness and the area of the foam,

respectively.

(Supplementary movie 8) Movie of a single collective rearrangement event seen during the relaxation process in a three-dimensional foam for ϕ = 0.28.

It is found that T1 events occur simultaneously. Playback is at 2x speed.

Definition of the characteristic length ξ of a relaxation event

A collective relaxation event involves multiple bubbles; there is a characteristic length associated with the event. Fig.S1 shows traces of the positions of the bubbles over time for ϕ_{2D} = **0.042**. Here, the characteristic length ξ of this event is defined as the furthest distance between two rearranged bubbles.

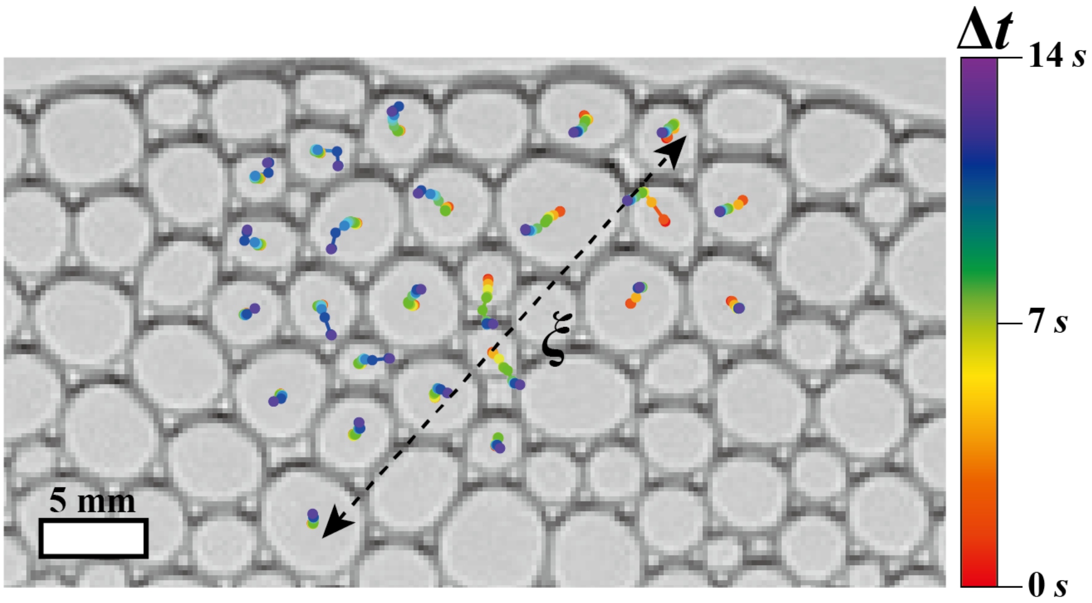

Fig. S1. Tracking of the positions of bubbles involved in a collective relaxation event for ϕ_{2D} = **0.042**. The characteristic length ξ of this event is defined as the furthest distance between two rearranged bubbles.

The effect of the anisotropy in surface tension on the outside of the foam

In order to confirm whether the anisotropy in surface tension on the outside of the foam affects the sequential rearrangement of bubbles, we investigate the case where the shape of the entire foam is elliptical. Figure S2 shows the image of an elliptical-shaped foam at ϕ_{2D} = 0.021. ***a*** and ***b*** represent

the lengths of the major and minor axes of the ellipse, respectively. Here, we introduce the flattening $\boldsymbol{f}$ of the foam, which is the compression factor of a circle along a diameter to form an ellipse, as defined by $\boldsymbol{f} = 1 - \boldsymbol{b}/\boldsymbol{a}$. Figure S3 shows $\boldsymbol{f}$ as a function of $\phi_{\mathbf{2D}}$. We find that $\boldsymbol{f}$ decreases as $\phi_{\mathbf{2D}}$ increases. Figure S4(a) and (b) show the mean relaxation time $\boldsymbol{\tau}$ and correlation length ξ of macroscopic rearrangement events averaged over several collective rearrangements as a function of $\phi_{\mathbf{2D}}$, respectively. We find that both $\boldsymbol{\tau}$ and ξ increase continuously as $\phi_{\mathbf{2D}}$ increases. This result is consistent with when the shape of the entire foam is circular, the main focus of this paper. Thus, we show that the anisotropy in surface tension on the outside of the foam does not affect collective rearrangement events.

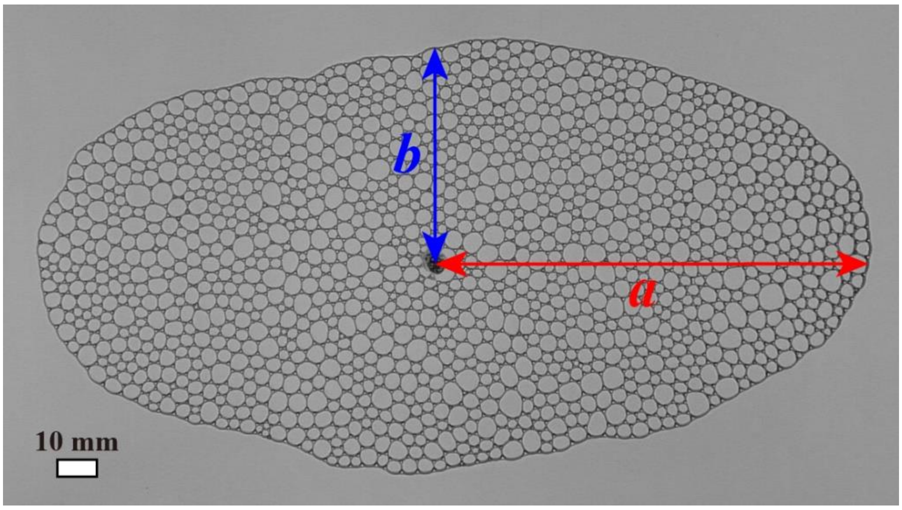

Fig. S2. Image of an elliptical foam at $\phi_{\mathbf{2D}} = \mathbf{0.021}$. $\boldsymbol{a}$ and $\boldsymbol{b}$ represent the lengths of the major and minor axes of the ellipse, respectively.

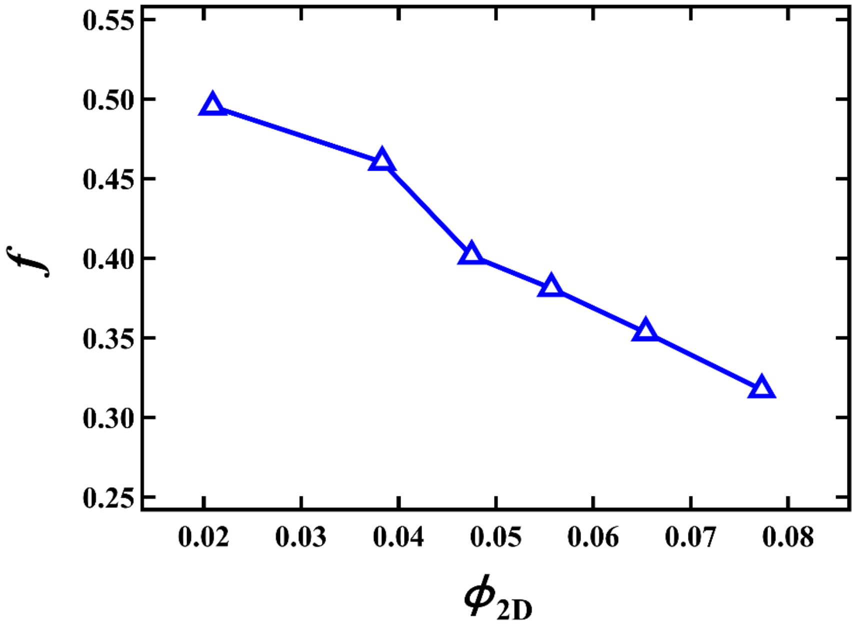

Fig. S3. Flattening f of the foam as a function of ϕ_{2D}. It is found that f decreases as ϕ_{2D} increases.

(a)

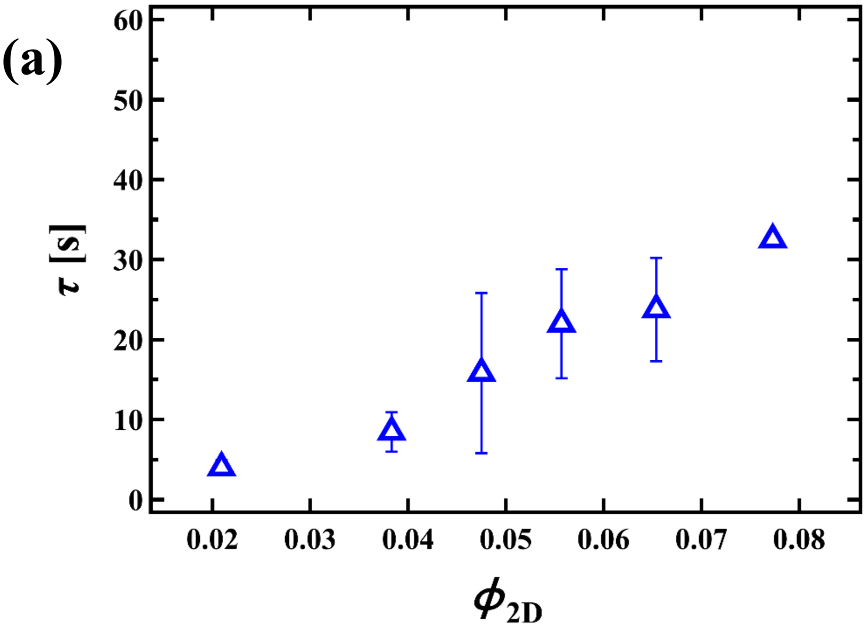

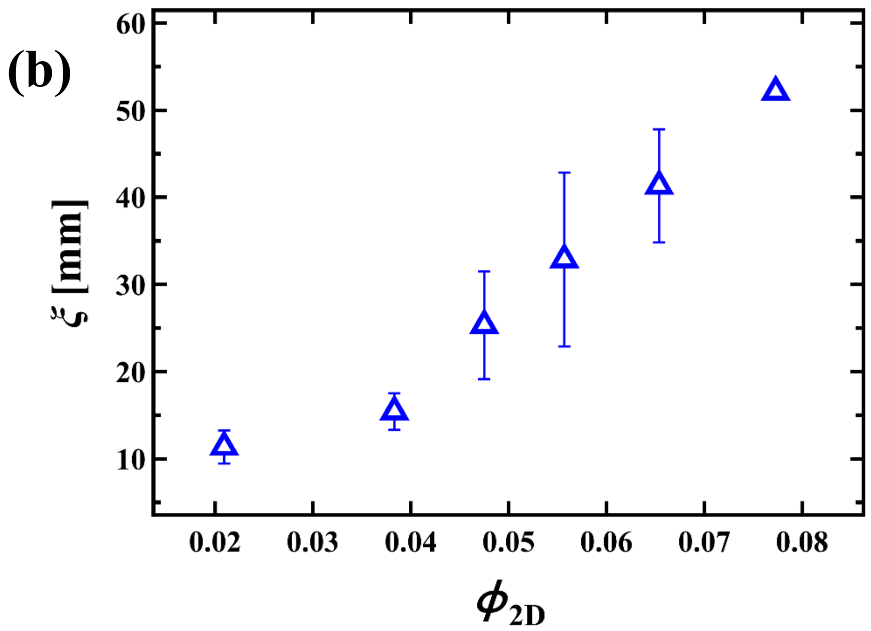

Fig. S4. (a) Mean relaxation time τ and (b) the correlation length ξ of macroscopic rearrangement events averaged over collective rearrangements as a function of ϕ_{2D}. It is found that both τ and ξ increase continuously as ϕ_{2D} increases.
